# Nationwide Survival Impact of Bevacizumab Under National Reimbursement for Advanced Cervical Cancer in South Korea

**DOI:** 10.3390/cancers18020346

**Published:** 2026-01-22

**Authors:** Junhwan Kim, Jieun Jang, Krishnansu S. Tewari, Kyung Su Kim, Hyun-Cheol Kang, Sokbom Kang

**Affiliations:** 1 Center for Gynecologic Cancer, National Cancer Center, Goyang 10408, Republic of Korea; junhwankimmd@gmail.com; 2 Division of Clinical Research, Research Institute, National Cancer Center, Goyang 10408, Republic of Korea; jjencc@ncc.re.kr; 3 Division of Gynecologic Oncology, Department of Gynecology and Obstetrics, University of California Irvine, Orange, CA 92868, USA; 4 Department of Radiation Oncology, Seoul National University Hospital, Seoul 03080, Republic of Korea; 5 Department of Radiation Oncology, Seoul National University College of Medicine, Seoul 03080, Republic of Korea

**Keywords:** Uterine Cervical Neoplasms, bevacizumab, chemoradiation therapy, reimbursement mechanisms, survival analysis

## Abstract

***Background:*** The aim of this study was to evaluate the effectiveness of bevacizumab in advanced cervical cancer (CC) patients using nationwide data after its inclusion in South Korea’s National Health Insurance (NHI), considering various clinicopathologic factors. ***Methods:*** This retrospective study analyzed 3869 advanced CC patients from South Korea’s cancer registry (2012–2019), alongside claims and death records (2012–2021). Among these 2792 patients diagnosed after bevacizumab’s NHI inclusion (August 2015), survival outcomes were compared between those receiving bevacizumab with platinum-based chemotherapy (*n* = 1787, 64.0%) versus chemotherapy alone (*n* = 1005, 36.0%). Overall survival (OS) was assessed using Cox proportional hazard regression with inverse probability of treatment weighting. ***Results:*** Following NHI coverage of bevacizumab, median OS increased from 1.5 to 2.5 years, and the 5-year OS rate increased from 25.6% to 41.4% (weighted hazard ratio [wHR], 0.63; 95% confidence interval [CI], 0.60–0.67). Among patients receiving bevacizumab, median OS was 2.6 years compared to 2.2 years for those not receiving bevacizumab, with 5-year OS rates of 42.0% and 40.2%, respectively (wHR, 0.84; 95% CI, 0.78–0.90). Subgroup analyses revealed that bevacizumab was associated with significantly better OS in patients with prior concurrent chemoradiation therapy (CCRT) history (wHR, 0.67; 95% CI, 0.61–0.75), regardless of histologic subtype (squamous cell carcinoma [SCC]: wHR, 0.69 [95% CI, 0.61–0.78] vs. non-SCC: wHR, 0.66 [95% CI, 0.55–0.79]). ***Conclusions:*** The national investment in the implementation of bevacizumab was associated with favorable survival outcomes in advanced CC patients. Particularly, bevacizumab showed pronounced survival benefit for patients with prior CCRT history, regardless of histologic subtype.

## Simple Summary

This study demonstrates that the addition of bevacizumab to chemotherapy improves overall survival in 2792 patients with advanced cervical cancer following the implementation of national insurance reimbursement, using data from the South Korean nationwide cohort. The findings suggest that bevacizumab offers a pronounced survival benefit, particularly for patients with a history of prior concurrent chemoradiation therapy, regardless of histologic subtype, highlighting the clinical value of reimbursement for high-cost novel cancer drugs in real-world practice.

## 1. Background

Cervical cancer (CC) is the fourth most common cancer in women worldwide, with an estimated 660,000 new cases and 350,000 deaths in 2022 [1]. While the global incidence of CC is declining due to the widespread implementation of human papillomavirus (HPV) vaccination and cervical screening programs, survival rates vary significantly depending on the disease stage at diagnosis [2]. For patients with early-stage CC, the 5-year survival rate is estimated to be higher than 90%, but the 5-year survival rate of advanced CC remains dismal and ranges from 14 to 60% due to limited curative options [3,4].

Over the past decade, the antiangiogenic agent bevacizumab has demonstrated promise in improving the overall survival (OS) of patients with advanced CC, as established by the pivotal GOG-240 trial [5,6]. Although the KEYNOTE-826 trial has since established pembrolizumab with platinum-based chemotherapy ± bevacizumab as the new standard of care, this regimen presents implementation challenges in Korea due to its substantial cost [7]. Since 2015, Korea’s National Health Insurance (NHI) program has been reimbursing bevacizumab with platinum-based chemotherapy to expand patient access for treatment of advanced CC. This reimbursement has established bevacizumab with chemotherapy as an important and commonly adopted first-line option for advanced CC in Korean real-world practice. However, Korea was not included in GOG-240, and the effectiveness of bevacizumab with chemotherapy had not been prospectively evaluated in Korean patients with advanced CC through phase 2 or phase 3 trials, with only a few retrospective studies available [8,9,10]. Therefore, questions remain about whether this substantial budgetary investment for bevacizumab has translated into clinically meaningful nationwide survival benefits for patients with advanced CC in Korea.

Thus, we aimed to evaluate the impact of the nationwide implementation of bevacizumab on survival outcomes in patients with advanced CC using a national registry, and to examine whether clinicopathologic factors such as prior concurrent chemoradiation therapy (CCRT), histology, and disease extent influenced its effectiveness.

## 2. Methods

### 2.1. Study Design

This study was conducted using a retrospective data cohort design based on the K-CURE platform, which provides public and clinical datasets of patients with cancer in Korea and is publicly available upon reasonable request via the K-CURE web page (https://k-cure.mohw.go.kr) [11]. We requested cancer registry data on patients with CC diagnosed from 2012 to 2019 in Korea and merged these data with death data provided by Statistics Korea and claims records provided by the health insurance review and assessment services of these patients from 2012 to 2021.

### 2.2. Eligibility of Study Subjects

**Figure 1:**
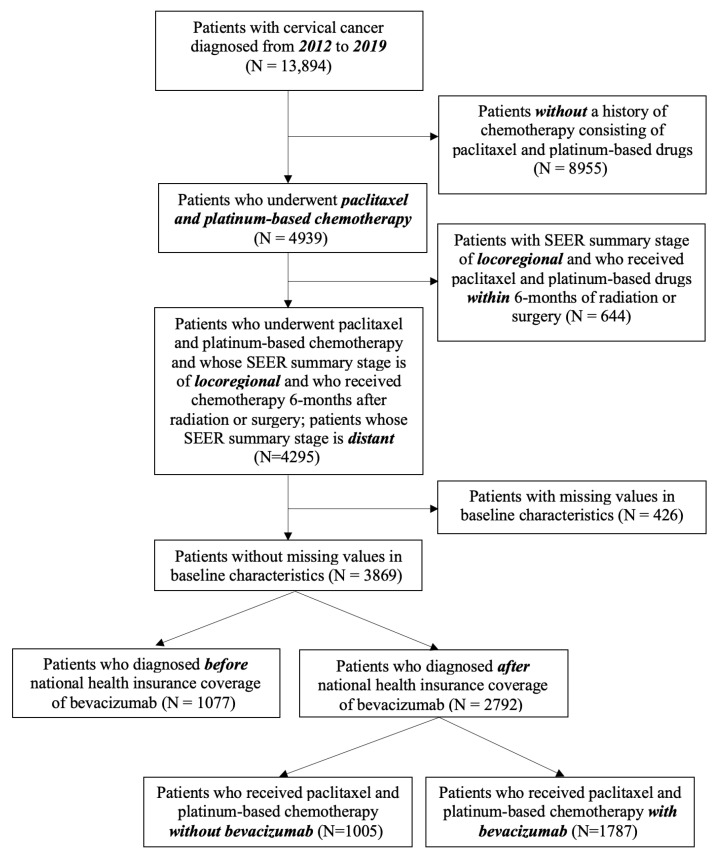
Flow diagram of the study. **Abbreviation:** SEER, The Surveillance, Epidemiology, and End Results.

We retrieved data from patients with CC, with the International Classification of Oncology 3rd code of ‘C53’, a malignant neoplasm of the cervix uteri, registered from 2012 to 2019 in Korea, and obtained 13,894 patients within that period. The flow diagram of this study is presented in Figure 1. After excluding patients with no history of platinum-based chemotherapy, those with a Surveillance, Epidemiology, and End Results (SEER) summary stage of locoregional at diagnosis who had received chemotherapy within 6 months after radiation or surgery, and those with missing baseline characteristic values, a total of 3869 patients were deemed eligible for this study. Of note, we excluded patients with locoregional disease who had received chemotherapy within 6 months after radiation or surgery, as they might have had an exceptionally poor prognosis that could not be generalized or received treatment not supported by current guidelines in real-world practice. After exclusion of these patients, 1077 were diagnosed before the national insurance coverage of bevacizumab (August 2015) in Korea, whereas 2792 were diagnosed after this coverage was implemented.

### 2.3. Operational Definitions of Cancer Stage and Cancer Treatment

We developed operational definitions to define cancer stage and cancer treatment. We utilized operational definitions to classify metastatic patients and those with recurrent or progressive cancer to define cancer treatments, including bevacizumab combination therapy and prior CCRT. We defined patients with recurrent or persistent disease as (1) patients with a SEER summary stage of locoregional at diagnosis who had claims records with medication codes corresponding to treatment with paclitaxel and platinum-based chemotherapy drugs or (2) patients with a SEER summary stage of distant at diagnosis, who have a claims record indicating radiotherapy or surgery at least 1 month before the first claim date with medication codes corresponding to paclitaxel and platinum-based chemotherapy drugs. For patients with metastatic disease, we defined them as patients with a SEER summary stage of distant at diagnosis, who do not have a claims record indicating radiotherapy or surgery at least 1 month before the first claim date for medication codes corresponding to paclitaxel and platinum-based chemotherapy drugs. Other detailed information regarding the operational definitions and the NHI fee codes of the procedures and medications used for the operational definitions is presented in Supplementary Tables S1 and S2, and Supplementary Figure S1.

### 2.4. Outcome Measures

The primary outcome was OS, defined as the time from initiation of first-line platinum-based chemotherapy with or without bevacizumab to death from any cause, comparing the periods before and after national reimbursement of bevacizumab. Secondary outcomes included (1) OS among patients who did and did not receive bevacizumab during the reimbursement period starting in August 2015 and (2) weighted hazard ratios (wHRs) for OS within prespecified subgroups stratified by bevacizumab exposure, SEER summary stage, tumor histology, and prior CCRT history.

### 2.5. Statistical Analysis

Baseline characteristics according to the concurrent use of bevacizumab and platinum-based chemotherapy drugs were compared via the chi-square test and *t*-test. The OS of patients with CC before and after the implementation of NHI coverage of bevacizumab and according to bevacizumab combination therapy are shown in Kaplan–Meier curves and were compared via the log-rank test. All-cause deaths among the subjects were identified using death data provided by Statistics Korea. The follow-up period for survival was calculated from the initiation of the concurrent prescription of platinum-based chemotherapy drugs and paclitaxel to the date of death or last follow-up (31 December 2021). To evaluate the impact of bevacizumab combination therapy on OS, we calculated the wHR via Cox proportional hazard regression with inverse probability of treatment weighting (IPTW). To validate the propensity score model, we assessed the balance of potential confounders between the exposure groups using standardized mean differences (SMDs). Post-weighting diagnostics confirmed adequate balance, with absolute SMDs for all covariates remaining below 0.1. The proportional hazards assumption was assessed by visually inspecting the Kaplan–Meier curves to ensure no crossing of survival lines occurred. We considered baseline characteristics, including age at diagnosis, income, SEER summary stage, histologic tumor type, and prior CCRT history, as explanatory variables in the binary logistic regression to estimate IPTW. Given the exploratory nature of the subgroup analyses, no formal multiplicity adjustment was applied; however, we interpreted *p*-values with caution and focused on effect sizes and confidence intervals (CI). All the statistical analyses were performed using SAS software (version 9.4; SAS Institute, Cary, NC, USA).

## 3. Results

### 3.1. Baseline Characteristics

Among 2792 patients with advanced cervical cancer (CC) who underwent paclitaxel and platinum-based chemotherapy, 1005 patients (36.0%) received chemotherapy without bevacizumab, and 1787 patients (64.0%) received bevacizumab (Figure 1). The incidence of nonsquamous cell carcinoma (non-SCC) was lower in patients who received bevacizumab (32.4% vs. 39.5%, *p* < 0.001), as shown in Table 1. Additionally, those treated with bevacizumab were more likely to have received prior CCRT (59.8% vs. 52.8%, *p* < 0.001) and had a higher incidence of distant disease (35.0% vs. 31.5%, *p* = 0.06). Recurrent or persistent disease was also less common in patients who received bevacizumab (78.5% vs. 82.2%, *p* = 0.02). Subgroup analysis of patients with locoregional disease revealed that prior CCRT was more common in the bevacizumab-treated group (72.5 vs. 58.7%, *p* < 0.0001), whereas non-SCC was more common in those who were not treated with bevacizumab (38.7% vs. 33.8%, *p* < 0.04), as presented in Supplementary Table S3. Among patients with distant disease, the proportion of the non-SCC histologic subtype was higher among patients treated with bevacizumab than in those who were not (58.7 vs. 41.3%, *p* < 0.001); however, the proportion of patients who received prior CCRT did not differ significantly between the groups (*p* = 0.25), as shown in Supplementary Table S4.

### 3.2. Comparison of Survival Before and After Bevacizumab Implementation

**Figure 2:**
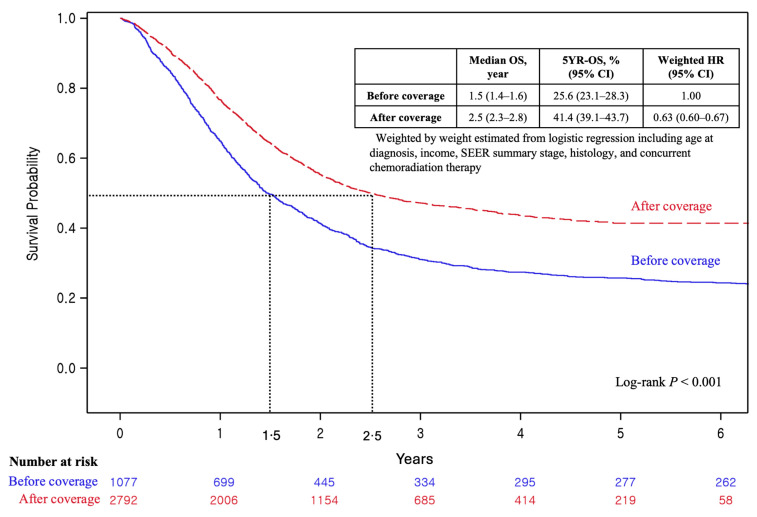
Overall survival of patients with advanced cervical cancer before and after the national health insurance coverage of bevacizumab (before and after August 2015). **Abbreviations:** CI, confidence interval; HR, hazard ratio; OS, overall survival; SEER, Surveillance, Epidemiology, and End Results.

Following the implementation of bevacizumab reimbursement by the Korean NHI starting in August 2015, its effectiveness was evaluated by comparing patient outcomes before (*n* = 1077) and after (*n* = 2792) the policy’s initiation (Figure 2). The median OS increased from 1.5 years (95% confidence interval [CI], 1.4–1.6) before the implementation of bevacizumab coverage to 2.5 years (95% CI, 2.3–2.8) after implementation. Similarly, the 5-year OS rate rose from 25.6% (95% CI, 23.1–28.3) to 41.4% (95% CI, 39.1–43.7), with a wHR of 0.63 (95% CI, 0.60–0.67) following implementation.

### 3.3. Effectiveness of Bevacizumab After Its Implementation Under National Reimbursement

The survival outcomes were further analyzed in patients diagnosed after the initiation of bevacizumab reimbursement (*n* = 2792), stratified by administration of bevacizumab (Table 2). The median OS was greater in patients treated with bevacizumab than in those not treated with bevacizumab (2.6 years [95% CI, 2.3–3.2] vs. 2.2 years [95% CI, 2.0–2.8]). The 5-year OS rates were 42.0% (95% CI, 39.0–45.1) for patients treated with bevacizumab and 40.2% (95% CI, 36.5–43.8) for those not treated with the drug (wHR, 0.84; 95% CI, 0.78–0.90).

### 3.4. Effectiveness of Bevacizumab According to Clinical Characteristics

**Figure 3:**
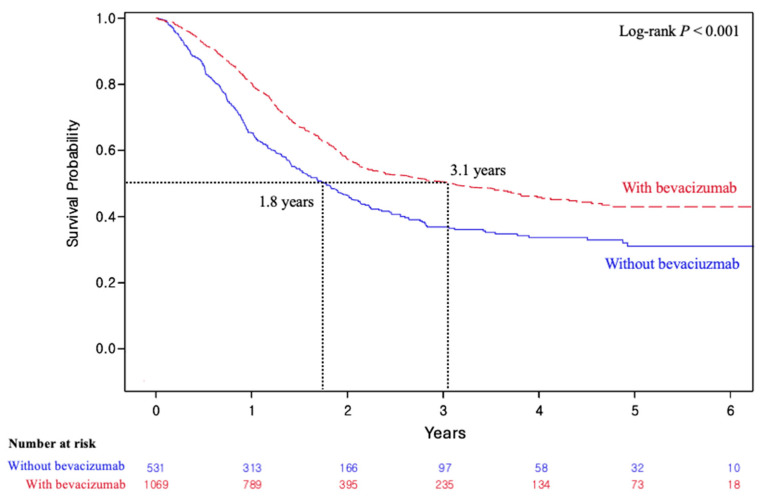
Overall survival of patients with advanced cervical cancer and history of prior concurrent chemoradiation therapy according to administration of bevacizumab.

The effectiveness of bevacizumab was also assessed by subgroup analyses according to the following clinical characteristics: stage, prior CCRT, and histology (Table 2). For stage, bevacizumab was associated with significantly better OS regardless of stage, with wHRs of 0.83 (95% CI, 0.76–0.92) and 0.80 (95% CI, 0.72–0.90) for locoregional (*n* = 1849) and distant disease (*n* = 943), respectively. In patients with a history of prior CCRT (*n* = 1600), bevacizumab was associated with improved OS (wHR, 0.67; 95% CI, 0.61–0.75), as demonstrated in Figure 3. Formal interaction analysis confirmed that the survival benefit was significantly more pronounced in this subgroup (*p* < 0.001). In contrast, patients without a history of prior CCRT (*n* = 1192) did not show significant OS benefit from bevacizumab (wHR, 1.05; 95% CI, 0.94–1.18). In terms of histology, only patients with the SCC histologic subtype (*n* = 1816) benefited from bevacizumab in terms of OS (wHR, 0.84; 95% CI, 0.77–0.92). Patients with non-SCC tumors (*n* = 976) did not show a statistically significant improvement in OS (wHR, 0.93; 95% CI, 0.82–1.05).

**Figure 4:**
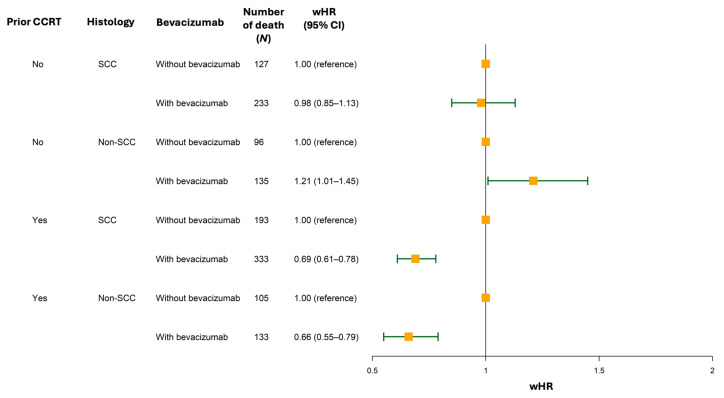
Forest plot presenting weighted hazard ratios for overall survival according to history of prior concurrent chemoradiation therapy and histology. **Abbreviations:** CCRT, concurrent chemoradiation therapy; CI, confidence interval; SCC, squamous cell carcinoma; wHR, weighted hazard ratio.

Since subgroup analyses revealed that prior CCRT and histology, unlike stage, were associated with varying survival outcomes after bevacizumab treatment, we performed additional analyses to investigate their concurrent effects on OS (Supplementary Table S5). For patients without a history of prior CCRT, bevacizumab was not associated with better OS regardless of histologic subtype (Figure 4, SCC: wHR, 0.98 [95% CI, 0.85–1.13] vs. non-SCC: wHR, 1.21 [95% CI, 1.01–1.45]). In contrast, for patients with a history of prior CCRT, bevacizumab was associated with improved OS regardless of histology (Figure 4, SCC: wHR, 0.69 [95% CI, 0.61–0.78] vs. non-SCC: wHR, 0.66 [95% CI, 0.55–0.79]).

## 4. Discussion

This study observed a nationwide trend of improved overall survival among Korean patients with advanced CC following the introduction of bevacizumab through NHI coverage. We also confirmed that patients treated with bevacizumab showed a statistically significant survival benefit compared to those who were not, based on nationwide data. Lastly, subgroup analyses demonstrated that the most pronounced survival benefit was observed in patients with a history of prior CCRT, regardless of histologic subtype.

Our study suggests that national efforts to reduce economic barriers to bevacizumab likely played a meaningful role in the observed survival outcomes in patients with advanced CC. Patients treated with bevacizumab in our study showed a median OS that surpassed the 16.8 months reported in the GOG-240 trial, which we attributed to the nature of real-world studies that include heterogeneous populations with less strict inclusion criteria [6]. However, advancements in treatment and palliative care may also have contributed to survival benefits, as evidenced by the higher median OS of patients who were not treated with bevacizumab after the reimbursement compared to those before the reimbursement (2.2 vs. 1.5 years). Off-label use of pembrolizumab monotherapy based on the KEYNOTE-158 trial may have contributed to improved OS in patients following the implementation of bevacizumab reimbursement, although real-world data showed only modest antitumor activity in patients with recurrent CC, comparable to that reported in previous clinical trials [12,13]. The gradual implementation of intensity-modulated radiation therapy and image-guided external radiation therapy, which reduce toxicity compared to three-dimensional conformal radiation therapy and allow patients to complete aggressive chemoradiation courses as planned, might be one of the contributing factors [14]. These techniques were reported to be used in approximately 60% of Korean centers for more than 50% of their clinical cases by 2019 [15]. In addition, the increasing use of stereotactic body radiotherapy, which delivers high-dose, precise radiation to metastases, may have contributed to the improved OS following the reimbursement of bevacizumab, as demonstrated in multiple Korean studies [16]. Furthermore, prophylactic irradiation to the para-aortic lymph node (LN) area (22.4% of Korean centers), routine implementation of LN boost (96.6% of Korean centers), and administration of ≥60 Gy to pelvic and retroperitoneal LNs (48% of Korean centers) in the reimbursement era may have also influenced the improvement in median OS [15]. Improvements in supportive care in Korea, including the reimbursement of prophylactic granulocyte colony-stimulating factor use to increase accessibility in 2015 and 2018, may also have contributed to improved OS [17].

Meanwhile, the weighted hazard ratio estimated after adjustment for potential confounders showed a statistically significant difference in this study, although the absolute difference in 5-year OS between patients who received bevacizumab and those who did not was modest. This discrepancy is likely attributable to baseline imbalances—namely, the unequal distribution of poor prognostic factors—between the comparison groups. Specifically, patients in the bevacizumab combination therapy group had a higher prevalence of non-squamous cell carcinoma, prior CCRT, and distant-stage disease. Consequently, while unadjusted 5-year OS did not fully account for these disparities, the weighted hazard ratio represents an effect estimate with relatively minimized bias. Furthermore, beyond the 5-year OS, the improvement in median OS provides compelling evidence of clinical significance. In our study, patients treated with bevacizumab demonstrated an absolute median survival gain of 0.4 years (approximately 4.8 months) compared to the untreated group (2.6 vs. 2.2 years). This gain aligns with and reinforces the clinical value established in the pivotal GOG-240 trial, which reported a median survival benefit of approximately 3.7 months.

It is noteworthy that a considerable number of patients with advanced CC were not exposed to bevacizumab in real-world settings, as observed in our study. We hypothesize that bevacizumab was avoided in these patients partly due to physicians’ apprehension regarding the relatively higher risk of perforation or fistula in patients with prior CCRT history, as emphasized in the literature [5,8,18,19,20]. Indeed, approximately half of the patients who were not treated with bevacizumab in this study were at high risk of perforation or fistula, as they had prior CCRT history. Nonetheless, it is worth highlighting that the survival benefit from bevacizumab was most pronounced in patients with prior CCRT history. Similarly, survival benefit was significant in patients who had received prior platinum-based CCRT in the GOG-240 subgroup analyses [5]. Several potential explanations can be proposed for this observation. First, radiotherapy-induced hypoxia may activate vasculogenic mimicry, thereby enhancing the effectiveness of antiangiogenic therapies in previously irradiated tissues [21]. Additionally, one study demonstrated that bevacizumab increased the effect of chemotherapy by reducing hypoxia-related treatment resistance and improving drug delivery [22]. In this biological regard, the most effective timing of bevacizumab for locoregional disease may be after radiotherapy, although prior irradiation history was significantly associated with a higher rate of bevacizumab-induced perforation or fistula [5,8,18,19,20]. Although retrospective studies of Korean patients with advanced CC reported a significantly higher rate of perforation or fistula in those who had undergone pelvic radiation therapy when bevacizumab was added, it is important to emphasize that, given the significant survival benefit observed in our study, this increased risk does not appear to compromise OS [8,20]. However, given the retrospective nature of this study, the survival benefit of bevacizumab for patients without prior CCRT history remains inconclusive due to multiple biases.

Another notable finding of our study is that bevacizumab was effective not only in patients with the SCC histologic subtype but also in those with non-SCC histologic subtypes, especially among those with a history of CCRT. In GOG-240, patients with the SCC histologic subtype derived a statistically significant benefit from bevacizumab, whereas this benefit was not confirmed in those with non-SCC subtypes because of the limited statistical power [5]. In contrast, our study provided sufficient statistical power because of the larger study population and greater proportion of patients with non-SCC histologic subtypes (32.4% vs. 11.9%). A previous retrospective study in Chinese patients with advanced CC also included a comparable number of patients with non-SCC histologic subtype (43%) as our study and demonstrated the survival benefit from bevacizumab, but survival analysis according to histologic subtype was not conducted [23]. On the other hand, we first identified an effective subpopulation of non-SCC patients who would benefit from bevacizumab. These findings provide strong evidence for predicting the therapeutic efficacy of bevacizumab in non-SCC patients.

Meanwhile, the improved OS from the bevacizumab group in our study was confirmed to be shorter when compared to the median OS (48.9 months for all-comers) of the current standard treatment for advanced CC, pembrolizumab with platinum-based chemotherapy ± bevacizumab from KEYNOTE-826 [24]. Although a head-to-head comparison is impossible, our result underscores that chemotherapy with bevacizumab should only be considered as a favorable first-line alternative when administration of pembrolizumab is limited in current practice. However, this limitation deserves attention in our real-world practice; the high cost of novel cancer therapies, including pembrolizumab, often poses a significant barrier to their widespread use and limits access to potentially life-extending treatments [7]. Financial constraints can lead to disparities in cancer care between patients who can afford treatment and those who cannot, with the economic burden of high-priced novel cancer treatments necessitating governmental financial support to ensure equitable access [25]. In Korea, the expansion of health insurance reimbursement for anticancer drugs is associated with significantly improved survival across multiple cancer types, suggesting that government intervention in healthcare financing can lead to better patient outcomes [26]. Similarly, the expansion of Medicaid is associated with improved timely treatment initiation and increased overall survival for multiple cancers, reinforcing the notion that financial assistance plays a crucial role in optimizing cancer care [27].

To address these challenges specifically, various financial and reimbursement models have been implemented globally, including non-risk-sharing models, performance-based models, risk-sharing models, alternative financing models, value-based models, and cost-reduction strategies [28]. Various countries have implemented strategies to improve access to novel cancer therapies through national reimbursement policies modifying those models. In Taiwan, a stepwise expansion of insurance coverage for gefitinib in non-small cell lung cancer from third-line to first-line treatment improved its overall accessibility [29]. China introduced a national-level price negotiation and insurance coverage policy for multiple targeted anticancer drugs in 2017, resulting in a 48.9% reduction in cost per defined daily dose of price-negotiated medications and a 143% increase in procurement volume [30]. For countries with limited national health insurance resources, innovative payment models have been proposed as alternatives. Belgium is exploring outcome-based spread payments for high-cost curative therapies like Advanced Therapy Medicinal Products [31]. Low-income countries are using the World Health Organization’s Essential Medicine List to reclassify a number of essential targeted therapies as general medicines, implementing tariff exemptions and local production policies to address price barriers [32]. These findings, along with our study, collectively underscore the necessity of financial support for high-cost novel cancer drugs to mitigate disparities in access to cancer treatments.

Our research underscores the urgent need for future studies aimed at enhancing accessibility to high-cost novel cancer drugs, in order to bridge the gap between the survival benefits observed in clinical trials and the actual outcomes in real-world practice. Reimbursement may offer a promising approach to improving access to high-cost novel cancer drugs. To maximize its effectiveness, it is essential to integrate both academic and administrative perspectives. From an academic perspective, quantitative evaluations using real-world data, as demonstrated in this study, are essential for performance-based policy decisions. Subgroup analyses that identify patient populations likely to experience superior treatment outcomes can support prioritized or differential reimbursement strategies in resource-limited settings. From an administrative perspective, establishing legal frameworks for risk-sharing agreements between pharmaceutical companies and insurers or national healthcare systems, coupled with income-based copayment structures, can help construct innovative payment models that balance access with fiscal sustainability. These comprehensive approaches are vital for developing reimbursement strategies that optimize patient outcomes and control healthcare expenditure by enhancing accessibility to novel cancer drugs.

Regarding the analytical strategy, while interrupted time-series (ITS) analysis is often employed to evaluate the population-level impact of policy interventions, we adopted a retrospective cohort design utilizing IPTW. An ITS approach effectively captures longitudinal trends and structural breaks following policy changes; however, it may be limited in adjusting for granular individual-level heterogeneity, such as specific histologic subtypes and prior treatment history, which are critical prognostic factors in advanced cervical cancer. Given our primary aim to evaluate the comparative effectiveness of bevacizumab in a real-world clinical setting rather than solely the policy’s net impact, the IPTW method was selected to rigorously balance baseline characteristics and minimize selection bias, although we acknowledge that unmeasured confounding remains a potential limitation inherent to observational designs.

Despite these rigorous analytical adjustments, our study has several limitations. First, the retrospective design of this study limited control over confounding variables and allowed for bias. Second, the study’s reliance on cancer registry data and claims records necessitated the use of operational definitions. Detailed clinical information was unavailable, specifically regarding performance status, comorbidities, exact histologic subtypes, biomarkers, and specific details on metastasis required to assess the burden of disease. For example, the absence of performance status data may introduce a “healthy user effect,” potentially overestimating the survival benefit if bevacizumab were preferentially prescribed to patients with better general health. Furthermore, data on off-label pembrolizumab use and adverse events, including bevacizumab-related perforations and fistulas, were lacking. These limitations may have resulted in unaccounted heterogeneity of the study population, leading to potential discrepancies between the assumed and actual clinical profiles. Third, as we could not capture detailed radiotherapy data such as total dose, fractionation, radiation field extent, and modality due to reimbursement-related data censoring, treatment heterogeneity might have influenced the radiotherapy outcomes and toxicity in the overall population or specific subgroups. Fourth, the subgroup analyses performed in this study were conducted without formal adjustment for multiple testing. While formal interaction tests were performed for primary stratification variables (e.g., prior CCRT history) and supported the observed heterogeneity, such tests could not be uniformly applied across all complex subgroups. Therefore, the possibility of type I error cannot be excluded, particularly in smaller subgroups. Accordingly, the findings from the subgroup analyses should be interpreted cautiously as exploratory and hypothesis-generating rather than as providing confirmatory evidence.

While keeping in mind the limitations stemming from the lack of specific clinical variables inherent to the base dataset, and interpreting the subgroup analyses with caution, we believe our study has the strength of providing robust evidence from a large nationwide cohort that bevacizumab significantly improves survival in patients with prior CCRT history, addressing previous safety concerns, and identifying effective subpopulations across histologic subtypes. These findings may support further national investment in the implementation of effective treatment strategies and offer insight into optimizing resource allocation for populations that derive the most significant benefit. Future large-scale studies are warranted to validate our findings and enable more precise analyses by overcoming the lack of feature collection through the incorporation of comprehensive clinical variables.

## 5. Conclusions

The nationwide implementation of bevacizumab-based chemotherapy by reducing economic barriers was followed by better survival outcomes in patients with advanced CC. In particular, bevacizumab showed survival benefits for patients with prior CCRT history, regardless of histologic subtype.

## Figures and Tables

**Table 1:** Baseline characteristics of the study population.

Characteristics	Bevacizumab Combination Treatment		*p* Value
	No *n* (%)	Yes *n* (%)	
**Age**			
*<70 years*	905 (90.1)	1620 (90.7)	0.60
*≥70 years*	100 (9.9)	167 (9.3)	
**Income**			
*<Median*	517 (51.4)	947 (53.0)	0.43
*≥Median*	488 (48.6)	840 (47.0)	
**Histology**			
*Squamous cell carcinoma*	608 (60.5)	1208 (67.6)	<0.001
Nonsquamous cell carcinoma *	397 (39.5)	579 (32.4)	
**CCRT**			
*No*	474 (47.2)	718 (40.2)	<0.001
*Yes*	531 (52.8)	1069 (59.8)	
**Stage**			
*Locoregional*	688 (68.4)	1161 (65.0)	0.06
*Distant*	317 (31.5)	626 (35.0)	
**Disease status**			
*Primary metastatic*	179 (17.8)	385 (21.5)	0.02
*Recurrent or persistent*	826 (82.2)	1402 (78.5)	

* This group includes adenocarcinoma, adenosquamous carcinoma, and others. Stage refers to the SEER summary stage. **Abbreviations:** CCRT, concurrent chemoradiation therapy; SEER, Surveillance, Epidemiology, and End Results.

**Table 2:** Overall survival by clinical characteristics.

Clinical Characteristics	Bevacizumab	Median (Year)	5-Year OS Rate % (95% CI)	Person-Year	Number of Surviving Patients *n* (%)	Number of Deaths *n* (%)	Weighted HR * (95% CI)
***Overall***	*Without bevacizumab*	2.2 (2.0–2.8)	40.2 (36.5–43.8)	2163	484 (48.2)	521 (51.8)	1.00
	*With bevacizumab*	2.6 (2.3–3.2)	42.0 (39.0–45.1)	3740	953 (53.3)	834 (46.7)	0.84 (0.78–0.90)
***Stage***		***Locoregional disease***					
	*Without bevacizumab*	4.5 (2.8–NR)	48.4 (43.8–52.8)	1580	388 (56.4)	300 (43.6)	1.00
	*With bevacizumab*	4.0 (3.3–NR)	47.1 (43.0–51.0)	2478	705 (60.7)	456 (39.3)	0.83 (0.76–0.92)
		***Distant disease***					
	*Without bevacizumab*	1.5 (1.3–1.8)	23.6 (18.2–29.4)	384	96 (30.3)	221 (69.7)	1.00
	*With bevacizumab*	1.7 (1.6–1.9)	31.9 (26.6–37.3)	1263	248 (39.6)	378 (60.4)	0.80 (0.72–0.90)
***CCRT***		***No Prior CCRT***					
	*Without bevacizumab*	4.0 (2.7–NR)	48.6 (43.5–53.5)	1121	251 (53.0)	223 (47.0)	1.00
	*With bevacizumab*	2.4 (2.1–2.8)	40.7 (36.3–45.0)	1567	350 (48.8)	368 (51.2)	1.05 (0.94–1.18)
		***Prior CCRT***					
	*Without bevacizumab*	1.8 (1.5–2.0)	31.0 (25.8–36.5)	943	233 (43.9)	298 (56.1)	1.00
	*With bevacizumab*	3.1 (2.4–3.8)	43.0 (38.9–47.1)	2174	603 (56.4)	466 (43.6)	0.67 (0.61–0.75)
***Histology***		***SCC***					
	*Without bevacizumab*	2.2 (1.9–2.9)	39.6 (35.0–44.3)	1322	288 (31.0)	320 (36.1)	1.00
	*With bevacizumab*	2.7 (2.3–3.6)	42.6 (39.0–46.2)	2581	642 (69.0)	566 (63.9)	0.84 (0.77–0.92)
		***Non-SCC***					
	*Without bevacizumab*	2.4 (1.9–3.0)	41.1 (35.3–46.7)	841	196 (38.7)	201 (42.9)	1.00
	*With bevacizumab*	2.5 (2.1–3.3)	40.5 (34.9–46.0)	1159	311 (61.3)	268 (57.1)	0.93 (0.82–1.05)

* Stage refers to the SEER summary stage. For each clinical characteristic, the hazard ratio was weighted using inverse probability treatment weighting, which was estimated via logistic regression. The model included age at diagnosis, income, histology, concurrent chemoradiation therapy, and the SEER summary stage and excluded the clinical characteristics that were evaluated. **Abbreviations:** CCRT, concurrent chemoradiation therapy; CI, confidence interval; HR, hazard ratio; NR, not reached; OS, overall survival; SEER, Surveillance, Epidemiology, and End Results.

## Data Availability

All data generated and analyzed during this study are included in this published article (and its Supplementary Materials). The datasets used and/or analyzed during the current study are available from the corresponding author on reasonable request. Proposals should be submitted to sokbom@ncc.re.kr within 12 months of the article’s publication.

## Supplementary Materials

The following supporting information can be downloaded at: https://www.mdpi.com/article/10.3390/cancers18020346/s1, Table S1. Operational definitions of cancer stage, cancer treatment, and comorbidities; Table S2. Korean National Health Insurance fee code for the procedures and medications used to define exposures and outcomes; Table S3. Baseline characteristics of patients with locoregional disease according to bevacizumab combination treatment; Table S4. Baseline characteristics of patients with distant disease according to bevacizumab combination treatment; Table S5. Overall survival according to prior chemoradiation and the impact of stage and histology; Figure S1. Operational definitions of recurrent, progressive and metastatic cancer.
