# Supplementary material for: Nationwide Survival Impact of Bevacizumab Under National Reimbursement for Advanced Cervical Cancer in South Korea
Source: Cancers (Basel). 2026 Jan 22;18(2):346. doi: 10.3390/cancers18020346 (PMC12838912; doi:10.3390/cancers18020346)

Nationwide Survival Impact of Bevacizumab under National Reimbursement for Advanced Cervical Cancer in South Korea

Supplementary Materials

Summary

- Number of tables: 5
- Number of figures: 1

Contents

Supplementary Table S1. Operational definitions of cancer stage, cancer treatment, and comorbidities ……………………………..…………………………………………………………………. P. 2

Supplementary Table S2. Korean National Health Insurance fee code for the procedures and medications used to define exposures and outcomes………………………………………………………………………………………… P. 3

Supplementary Table S3. Baseline characteristics of patients with locoregional disease according to bevacizumab combination treatment………………………………………………………………. P. 4

Supplementary Table S4. Baseline characteristics of patients with distant disease according to bevacizumab combination treatment……………………………………..…………………………... P. 5

Supplementary Table S5. Overall survival according to prior chemoradiation and the impact of stage and histology…………………………………..…………………………………………………...... P. 6

Supplementary Figure S1. Operational definitions of recurrent, progressive and metastatic cancer. ……………………………………..………………………………………………………………. P. 8

Supplementary Table S1. Operational definitions of cancer stage, cancer treatment, and comorbidities

Variables	Operational definition	Data source
Bevacizumab combination therapy	Having a claims record with a medication code corresponding to simultaneous treatment with bevacizumab, platinum-based drugs, and paclitaxel	Claims record by HIRA
Prior concurrent chemoradiotherapy	Having a claims record with a medication code and procedure code corresponding to platinum-based chemotherapy drugs or radiation treatment ***prior to*** the first claim date of simultaneous chemotherapy of paclitaxel and platinum-based drugs	Claims record by HIRA
Subsequent concurrent chemoradiotherapy	Having a claims record with a medication code and procedure code corresponding to platinum-based chemo drugs or radiation treatment ***after*** the first claim date of simultaneous chemotherapy with paclitaxel and platinum-based drugs	Claims record by HIRA
Patients with recurrent or progressive disease	1) Patients with a SEER summary stage of localized or regional at diagnosis (*the first-line treatment is concurrent chemoradiotherapy in Korea for these group*), and having a claims record with medication codes corresponding to treatment with paclitaxel and platinum-based chemotherapy drugs, or 2) Patients with a SEER summary stage of distant at diagnosis, who have a claims record indicating radiation therapy or surgical treatment at least 1 month before the first claim date with medication codes corresponding to paclitaxel and platinum-based chemotherapy drugs	Claims record by HIRA
Metastatic patients	Patients with a SEER summary stage of distant at diagnosis, who do not have a claims record indicating radiation therapy or surgical treatment at least 1 month before the first claim date with medication codes corresponding to paclitaxel and platinum-based chemotherapy drugs	Claims record by HIRA

Abbreviations: HIRA, Health Insurance Review and Assessment Service

Supplementary Table S2. Korean National Health Insurance fee codes for procedures and medications used to define exposures and outcomes

Class	Code	Description of procedures, medications, and diagnosis
Procedure	Cervical cancer-related surgery	
	R4073	Extirpation of Vaginal Tumor-Malignant(Simple)
	R4074	Extirpation of Vaginal Tumor-Malignant(Radical)
	R4130	Subtotal Hysterectomy
	R4143	Hysterectomy(With Lymphadenectomy)-Simple
	R4144	Hysterectomy(With Lymphadenectomy)-Complex
	R4145	Hysterectomy(Without Lymphadenectomy)-Simple
	R4146	Hysterectomy(Without Lymphadenectomy)-Complex
	R4183	Correction of Uterine Inversion-Total Hysterectomy
	R4202	Vaginal Total Hysterectomy
	R4203	Vaginal Total Hysterectomy with A and P Repair
	R4221	Total Hysterectomy
	R4223	Subtotal Hysterectomy
	R4482	Evacuation of Hydatidiform Mole-Total Hysterectomy
	Radiotherapy	
	HD051	Low energy teletherapy, single port.
	HD052	Middle energy teletherapy, single port.
	HD053	High energy teletherapy, single port.
	HD054	Low energy teletherapy, parallel opposed ports.
	HD055	Middle energy teletherapy, parallel opposed ports.
	HD056	High energy teletherapy, parallel opposed ports.
	HD057	Low energy rotational irradiation
	HD058	Middle energy rotational irradiation
	HD059	High energy rotational irradiation
	HD061	3-Dimensional conformal therapy
	HD080	Brachytherapy
	HD081–HD082	Intracavity therapy, high dose rate
	HD083–HD084	Intracavity therapy, low dose rate
	HD085–HD086	Interstitial/intraluminal therapy, high dose rate
	HD087–HD088	Interstitial/intraluminal therapy, high dose rate
	HD121	Proton therapy
	HZ271	Intensity modulated radiation therapy
Medication	Platinum-based chemotherapy drugs	
	1345	Cisplatin
	1237	Carboplatin
	2078	Paclitaxel
	5543	Bevacizumab

Supplementary Table S3. Baseline characteristics of patients with locoregional disease according to bevacizumab combination treatment

**Characteristics**	**Bevacizumab combination treatment**		
	**No** N (%)	**Yes** N (%)	***p* value**
**Age**			
<70 years	628 (91.3)	1,063 (91.6)	0.84
≥70 years	60 (8.7)	98 (8.4)	
**Income**			
<Median	343 (49.9)	609 (52.5)	0.28
≥Median	345 (50.1)	552 (47.5)	
**Histology**			
Squamous cell carcinoma	422 (61.3)	768 (66.2)	<0.04
Nonsquamous cell carcinoma*	266 (38.7)	393 (33.8)	
**CCRT**			
No	284 (41.3)	319 (27.5)	<0.0001
Yes	404 (58.7)	842 (72.5)	

**This group includes adenocarcinoma, adenosquamous carcinoma, and others.*

Abbreviations: CCRT, concurrent chemoradiation therapy; SEER, Surveillance, Epidemiology, and End Results

Supplementary Table S4. Baseline characteristics of patients with distant disease according to bevacizumab combination treatment

**Characteristics**	**Bevacizumab combination treatment**		**p value**
	**No**<br>**N (%)**	**Yes**<br>**N (%)**	
**Age**			
<70 years	277 (87.4)	557 (89.0)	0.47
≥70 years	40 (12.6)	69 (11.0)	
**Income**			
<Median	174 (54.9)	338 (54.0)	0.79
≥Median	143 (45.1)	288 (46.0)	
**Histology**			
Squamous cell carcinoma	186 (58.7)	440 (70.3)	<0.001
Nonsquamous cell carcinoma*	131 (41.3)	186 (29.7)	
**CCRT**			
No	190 (59.9)	399 (63.7)	0.25
Yes	127 (40.1)	227 (36.3)	

**This group includes adenocarcinoma, adenosquamous carcinoma, and others.*

Abbreviations: CCRT, concurrent chemoradiation therapy; SEER, Surveillance, Epidemiology, and End Results

Supplementary Table S5. Overall survival according to prior chemoradiation and the impact of stage and histology

Clinical Characteristics	Bevacizumab	Median (Year)	5-year OS rate % (95% CI)	Person-year	Number of surviving patients N (%)	Number of deaths N (%)	Weighted HR (95% CI)
Stage				***No Prior CCRT***			
	**Locoregional disease**						
	Without bevacizumab	*NR*	64.8 (58.2–70.5)	853	194 (68.3)	90 (31.7)	1.00
	With bevacizumab	*NR*	53.9 (46.7–60.5)	727	204 (60.4)	115 (36.0)	1.32 (1.09–1.61)
	**Distant disease**						
	Without bevacizumab	1.6 (1.3–1.9)	24.4 (17.6–31.8)	368	57 (30.0)	133 (70.0)	1.00
	With bevacizumab	1.7 (1.6–2.0)	31.9 (26.6–37.3)	839	146 (36.6)	253 (63.4)	0.85 (0.74–0.98)
				Prior CCRT			
	**Locoregional disease**						
	Without bevacizumab	2.0 (1.7–2.5)	34.1 (27.8–40.5)	727	194 (48.0)	210 (52.0)	1.00
	With bevacizumab	3.6 (2.8–4.7)	44.3 (39.4–49.1)	1,750	501 (59.5)	341 (40.5)	0.66 (0.59–0.74)
	**Distant disease**						
	Without bevacizumab	1.3 (1.0–1.5)	22.4 (13.9–32.1)	216	39 (30.7)	88 (69.3)	1·00
	With bevacizumab	1.7 (1.4–2.3)	38.2 (30.8–45.6)	423	102 (44.9)	125 (55.1)	0.73 (0.61–0.89)
Histology				***No Prior CCRT***			
	SCC						
	Without bevacizumab	3.8 (2.5–*NR*)	49.2 (42.6–55.5)	709	143 (53.0)	127 (47.0)	1.00
	With bevacizumab	2.5 (2.2–3.8)	43.1 (37.8–48.3)	1,062	235 (50.2)	233 (49.8)	0.98 (0.85–1.13)
	Non-SCC						
	Without bevacizumab	4.0 (2.3–*NR*)	47.6 (39.6–55.2)	512	108 (52.9)	96 (47.1)	1.00
	With bevacizumab	2.1 (1.8–2·7)	36.0 (28.5–43.5)	504	115 (46.0)	135 (54.0)	1.21 (1.01–1.45)
				Prior CCRT			
	SCC						

Without bevacizumab	1.7 (1.4–2.1)	30.4 (23.9–37.2)	613	145 (42.9)	193 (57.1)	1.00
With bevacizumab	3.1 (2.1–3.8)	42.2 (37.3–47.0)	1,518	407 (55.0)	333 (45.0)	0.69 (0.61–0.78)
Non-SCC						
Without bevacizumab	1.8 (1.4–2.3)	33.0 (24.9–41.2)	329	88 (45.6)	105 (54.4)	1.00
With bevacizumab	3.0 (2.4–*NR*)	44.5 (36.7–52.1)	655	196 (59.6)	133 (40.4)	0.66 (0.55–0.79)

[*] Stage refers to the SEER summary stage.

[**] For each clinical characteristic, the hazard ratio was weighted using inverse probability treatment weighting, which was estimated via logistic regression. The model included age at diagnosis, income, histology, concurrent chemoradiation therapy, and the SEER summary stage and excluded the clinical characteristics that were evaluated.

Abbreviations: CCRT, concurrent chemoradiation therapy; CI, confidence interval; HR, hazard ratio; NR, not reached; OS, overall survival; SEER, Surveillance, Epidemiology, and End Results

Supplementary Figure S1. Operational definitions of recurrent, progressive and metastatic cancer

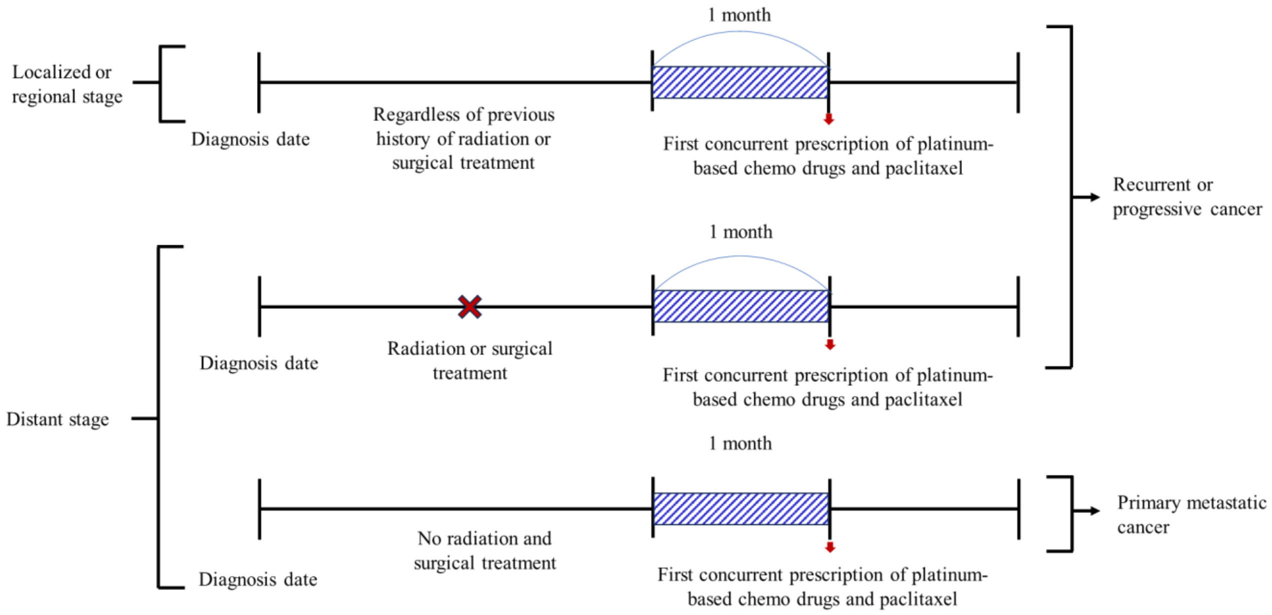

Supplement: Supplementary file 1 [file cancers-18-00346-s001.zip › cancers-4043253-supplementary.pdf]
